# Educating teachers toward immigrant empowerment and liberation in the United States and Chile: A funds of knowledge perspective

**DOI:** 10.1002/ajcp.12797

**Published:** 2025-02-20

**Authors:** Ana Christina da Silva Iddings, Macarena Lamas, Carola Lourdes Aravena Rojas, Katherine Malhue Vasquez

**Affiliations:** ^1^ Vanderbilt University Nashville Tennessee USA; ^2^ Universidad Austral de Chile Valdivia Chile; ^3^ Pontificia Universidad Católica de Valparaíso Valparaíso Chile

**Keywords:** early childhood teacher education, immigrant empowerment, immigrant family and community engagement

## Abstract

This article examines how university‐based teacher education programs in diverse historical, sociocultural, and political settings in the U.S. and in Chile, served to foster immigrant empowerment and liberation. Using a *Funds of Knowledge* approach, the study analyzed the educational practices of migrant families and their integration into early childhood teacher education curricula in both countries. Ethnographic methods, including teacher interviews, household visits, and case studies, revealed that engaging with immigrant families fostered critical thinking among teachers, deepened their respect for the families' values and aspirations, and highlighted the rich educational resources within immigrant households. The study underscores the importance of recognizing and incorporating these practices to support young immigrant children's development. However, it also stresses the urgent need to address systemic oppression and entrenched colonial‐racial ideologies that persist in educational systems in both contexts.

## Highlights

- Funds of Knowledge create opportunities for inclusivity and challenge deficit‐based discourses.
- University‐based teacher education programs must prepare teachers to challenge systemic inequalities.
- Funds of Knowledge have shown to be useful and relevant in various parts of the U.S. and the world.

## INTRODUCTION

Migrants are moving across the globe at an unprecedented rate due to factors such as persecution, war, conflict, violence, poverty, and human rights violations. There is little evidence to suggest that these movements will slow down in the future (International Organization for Migration, 2024). Considering this ongoing trend, this article examines a collaborative initiative between universities in the United States and Chile. Specifically, it addresses the question: *How can university‐based teacher education foster the empowerment and liberation of immigrant families within diverse historical, sociocultural, and political contexts?*

Our research employed a Funds of Knowledge approach (González et al., 2005), which offers a robust theoretical framework and a rigorous methodological lens for understanding the educational practices of immigrant families. This approach also provides a basis for examining how these practices might inform curriculum development in early childhood education within the United States and Chile. To pursue these aims, we conducted ethnographic research in both countries, incorporating interviews with preservice and in‐service teachers, classroom observations, and home visits with immigrant families. Through our combined efforts, we seek to challenge the deficit‐oriented perspectives often associated with immigrant families in both national contexts. By examining their goals, practices, and tools, we highlight the rich resources and strategies these families employ to navigate educational systems.

Our work is at the intersection of education, psychology, critical studies, applied linguistics, and anthropology. Within this interdisciplinary space, we focus on narratives, lived experiences, histories, cultural practices, and sociopolitical contexts, using these as lenses to theorize and inform educational research. We argue that preparing educators to work with immigrant children and families in both the U.S. and Chile requires a comprehensive engagement with these complexities. This preparation must resist deficit‐oriented, reductive, and essentialized perspectives, which often neglect or oversimplify students' lived experiences, cultural contexts, and social realities. To do so, educators must prepare teachers to move beyond efficiency‐driven educational models that ignore the nuanced realities of immigrant communities.

We develop our argument in the contexts of the United States and Chile through the following key points: (I) an elaboration on the historical and sociopolitical circumstances affecting immigrant students and families, (II) an examination of the impact of deficit‐based discourses on the educational experiences of immigrant students and families, (III) a proposal for a more nuanced conceptualization of “culture” and “critical interculturality,” (IV) a discussion of Funds of Knowledge (González et al., 2005) as a foundational concept for fostering criticality in teacher education, (V) an analysis of literature highlighting the importance of bridging family‐school educational practices, and (VI) comparative illustrations from our respective research on the application of Funds of Knowledge in teacher education programs.

### The historical and sociopolitical context of immigrant students in the U.S. and Chile

To comprehend the unique patterns and public responses to immigration in the United States and Chile, examining their distinct historical contexts is helpful.

#### Immigration in the United States

Immigration has been a continuous feature of U.S. history, marked by two major epoch‐making periods: the Mass Immigration Era (1880–1924), from southern and eastern Europe, and the post‐1965 Immigration Surge, primarily from Latin America and Asia (Portes & Rumbaut, 2024). Each period brought more than 25 million immigrants, and the current wave shows no signs of abating. From the colonial era to the present, immigration policy and public attitudes toward immigrants have fluctuated dramatically, oscillating between openness and hostility. These shifts often aligned with economic trends: during periods of rapid economic growth, such as the Industrial Revolution or post‐war booms, immigrants were welcomed to fill labor demands. However, economic downturns often incited fear and exclusionary policies aimed at limiting immigration. This pattern reflects the dual forces driving U.S. immigration policy—economic necessity and fear of the foreigner.

Since 1783, more than 86 million people have legally immigrated to the U.S., yet the legal frameworks governing their arrival have changed significantly over time. Despite these shifts, immigration has remained a contentious political issue. Public discourse often invokes the notion that the United States is a “nation of immigrants.” However, historian Roxanne Dunbar‐Ortiz (2021) argues that this ideology is misleading and harmful. It obscures America's foundational history of settler colonialism, genocide, slavery, and structural inequality—legacies that persist today. She contends that this narrative emerged during the 1960s as a strategic response by the ruling class to demands for decolonization, reparations, and social justice. The portrayal of America as a land of opportunity, built by immigrants, promotes a sanitized history of progress that conceals the violent displacement of Indigenous peoples and the exploitation of enslaved Africans. While some Americans are immigrants or descendants of immigrants, others are descendants of settlers who came to colonize and displace Indigenous populations. Still others are descendants of individuals forcibly brought to the U.S. through enslavement. Dunbar‐Ortiz asserts that recognizing this diversity of experiences is essential for a truthful understanding of America's history and its ongoing struggles with inequality and exclusion.

Conflicting visions and piecemeal legislation have left the United States with an outdated and incoherent immigration system, driven by obsolete policy objectives and controlled by the executive branch. According to a Pew Research Center (2024) report, approximately 47.6 million immigrants currently reside in the U.S., including 11 million undocumented individuals. About 5 million are enrolled in U.S. elementary and secondary schools. A combination of push and pull factors sustain a steady flow of migration to the U.S., including war, extreme violence, family reunification efforts, and economic opportunities (Moslimani & Passel, 2024). While anti‐immigration laws have long been a part of U.S. history, the past decade, particularly during the Trump administration, marked an even harsher turn. Militarized borders, excessively restrictive immigration policies, and family separations at the border have created lasting, harrowing images in the public consciousness. These measures have polarized public discourse, stirred chaos in the media, and deepened confusion surrounding immigration issues in the U.S. Additionally, intensified deportation measures and other severe tactics—such as racial profiling (“show me your papers” laws), and raids on homes, workplaces, and places of worship—have instilled a pervasive sense of fear and intimidation among long‐established immigrant families. In the 2024 presidential election, immigration and border security remained key issues influencing voter decisions. Many Americans expressed concerns that the recent influx of migrants has negatively affected the economy, crime rates, national security, and government spending (Parti & Hackman, 2024).

#### Immigration in Chile

Migration has been a constant and structural phenomenon throughout Chile's history as a young republic. However, recent socio‐political tensions and economic downturns across Latin America have reshaped immigration patterns to Chile, prompting new public responses. Over the past decade, most new arrivals have come from Venezuela, Peru, Haiti, and Colombia. In response, Chile has enacted increasingly restrictive immigration laws, heightening the likelihood of deportation. To address the influx, an Interministerial Committee was established in May 2022 to refine immigration legislation, and by December 2022, the National Migration Service formed a civil society advisory board. Additionally, the release of Chile's first National Immigrant Survey revealed concerning trends in the integration and living conditions of migrant groups. These issues were further underscored in April 2023, when hundreds of Venezuelan migrants were stranded at the Peru‐Chile border, with many eventually repatriated to Venezuela (Chile National Migration Service, 2024).

Early migration to Chile was historically characterized by state‐sponsored efforts to attract European settlers. These settlers were encouraged to populate the vast southern territories, traditionally inhabited by the Mapuche people, under the pretext of fostering industrial growth and enhancing the nation's “cultural and social wealth” (Cano & Soffia, 2009). This state‐driven ideal of whiteness was instrumental in creating a homogenizing, hegemonic discourse that aligned with racial superiority narratives (Tijoux and Córdova, 2015).

In recent years, Chile's economic growth, democratic stability, and restricted immigration policies in Europe and the United States have made it an attractive destination for migrants from Latin America and the Caribbean. However, these newer migration patterns have also been accompanied by historical and systemic issues of discrimination, racism, xenophobia, and social exclusion (Cociña‐Cholaky, 2020). These challenges echo earlier processes of exploitation and subjugation, including the internment of Afro‐descendant slaves (Duconge & Lube Guizardi, 2014) and the colonial oppression of Indigenous populations.

This historical legacy has contributed to the construction of a racialized image of the Latin American migrant, framed by stereotypes and prejudices that, as Tijoux (2013) argues, emphasize the differentiation between the white European and the ethnic ancestral. Within this duality, Latin American migrants are often perceived as racialized, deficient, and inferior individuals. These migrants are drawn by the promise of greater stability, improved job opportunities, and better living conditions (Gissi et al., 2021). While Chile's migrant population (7.8%) remains below the OECD average of 14.3%, it has grown persistently. From 465,319 migrants in 2015, the number rose to 1.5 million by 2021, making Chile the third‐ranking OECD country in terms of immigration growth during this period (Instituto Nacional de Estadísticas INE & Departamento de Extranjería y Migración DEM, 2021). These respective histories of immigration, both in the U.S. and Chile, along with outdated immigration laws and policies in both countries, have entered the public discourse and produced a narrative of the educational deficit with respect to the academic potential and capabilities of immigrant children.

### The impact of deficit‐based discourses on the educational experiences of immigrant students and families

When immigrant children cross the threshold of the preschool or kindergarten door, within the institutional framework of schools, they begin to author their sense of self (Da Silva Iddings, 2017). In both the United States and Chile, dehumanizing, colonial‐minded policies and related practices have created harmful narratives that often lead immigrant and minoritized populations to internalize identities that do not serve them. Speaking of the U.S. context, González (2016) argues, “immigrant children and families in the United States are forced to read, to decode the world, through a narrative of illegitimacy, of not being authorized, of not belonging, regardless of credentials, regardless of legal authorization, and regardless of permanence of place or history. And these are the stories that our immigrant children have woven into their sense of place‐making, their sense of belonging/not belonging, and their sense of citizenship/not citizenship” (p. 3). Similarly, in Chile, deficit discourses around education are also widely present in remnant colonial structures, curricula, and policies. There, as in the United States, the Western educational architecture (where scientific knowledge is represented as legitimate knowledge) is closely aligned with principles of school performance based on standardized, linear, and bottom‐up progress. Consequently, as in the United States, responses to cultural, ethnic, and linguistic diversity in Chilean schools have been based on deficit‐based perspectives. These perspectives refer to an individual's physical, mental disability or situation of linguistic, social, and/or cultural differences as a “disadvantage” because they cause a deviation from the norm. Such narratives portray these populations as people lacking abilities, skills, and social and cultural resources that allow them to develop on equal terms in a country perceived as homogeneous (González, 2016). Consequently, these social categories become general and universal referents to define the identity of migrant children in schools, who are also classified as a “vulnerable population” (Parra Muñoz et al., 2020). Thus, assimilationist responses are reinforced in schools to achieve optimal academic performance and homogenization, putting the focus on the results obtained in standardized tests (Jiménez‐Vargas & Montecinos‐Sanhueza, 2019).

As a result, schools have been compelled to redesign their educational practices ad hoc and improvised (Jiménez‐Vargas et al., 2020). Although individual schools' practices, procedures, and protocols differ in their purposes, pedagogical functions, and levels of sophistication, they often reveal tensions and contradictions in addressing cultural diversity (Valdés et al., 2022). Recent research indicates that many Chilean teachers view cultural and linguistic diversity as a barrier to educational processes, frequently resorting to assimilationist approaches to manage it (Stang‐Alva et al., 2021). In many instances, cultural and linguistic diversity is either rendered invisible or subsumed under the dominant “norm,” preventing significant changes in teaching methodologies (Jiménez & Fardella, 2015).

An aggravating factor in effectively addressing cultural and linguistic diversity in schools and classrooms, both in Chile and the United States, is the inadequate preparation of teachers and education professionals in university programs. These programs often fail to equip educators with a critical perspective necessary to challenge deficit‐oriented narratives. In both countries, the primary challenge lies in transitioning from a traditional teaching and learning model—one rooted in a homogenizing, deficit‐based approach oriented toward standardization (Arnaiz, 2003; Rodríguez‐Revelo, 2017)—to a model that embraces heterogeneity, prioritizes diversified teaching methods, and fosters nuanced understandings of cultural and linguistic diversity. To achieve this, the concepts of Critical Interculturality and Funds of knowledge (González et al., 2005) offer a valuable foundation for designing teacher education programs. These frameworks can help prepare educators to better address the educational needs and empowerment of culturally and linguistically diverse immigrant students and their families and communities.

### Complexifying the concept of culture and understanding critical interculturality

Critical interculturality is an approach to culture that does not frame cultural diversity as the central issue but rather focuses on the structural‐colonial‐racial problem. This problem underpins the creation of differences, perpetuates inequalities and inequities in colonized regions, and marginalizes, denies, or even erases alternative forms of knowledge and worldviews. Portuguese sociologist De Sousa Santos (2018) encapsulates these processes as a “massive *epistemicide*,” rooted in the dominance of modern Western science and its prioritization above all other forms of understanding. He further argues that “from the affirmation that the indigenous understanding of the world far surpasses the Western understanding of the world, is from where the epistemologies of the Global South emerge, from the vindicative struggles of all those sectors, populations, communities, nations that have systematically suffered the injustices, oppression, and destruction caused by capitalism, colonialism, and patriarchy” (p. 212).

In this light, critical interculturality goes beyond the superficial exchange of practices and values between cultures. It emphasizes the relational dimension and resists erasing the conflicts and power dynamics that underpin these interactions. This framework starkly opposes the colonizing tendencies of functional interculturalism (Tubino, 2004), a concept prevalent in official discourses. Functional interculturalism recognizes diversity and cultural differences, encourages dialogue, and promotes coexistence and tolerance but operates within the existing power structures without challenging the roots of asymmetries or hegemonic power's social and structural inequalities. This approach aligns with the current neoliberal economic and political model, managing and producing differences while upholding a hierarchical national order. Consequently, cultural diversity is stripped of its intrinsic value and significance, reduced to a superficial esthetic under a glossy veneer. Within this framework, monocultural and standardized educational curricula persist, reinforcing prescriptive and exclusionary norms.

In response to these challenges, we argue that teacher preparation curricula must undergo significant reforms to abandon deficit‐based perspectives. Instead, they should embrace reflective teaching practices aimed at addressing inequalities, celebrating diverse identities, and combating prejudice. Such reforms would pave the way for critical‐intercultural and antiracist education (Valdés et al., 2022). To achieve this, we propose incorporating the Funds of Knowledge framework into teacher education. This approach draws on the lived experiences of local communities, enabling the development of new epistemic configurations that revalue the knowledge, practices, and emotions of culturally oppressed groups—elements often disregarded and rendered invisible by the persistence of colonial ideologies.

### Funds of knowledge as a fundamental concept to promote criticality in teacher education

For over three decades, researchers such as Moll and González (1994), Vélez‐Ibáñez and Greenberg (1992), and González et al. (2005) have developed and expanded the concept of Funds of Knowledge, which highlights the rich knowledge and skills embedded within local households and families, particularly in the U.S. In their foundational work, González et al. (2005) identified historical and cultural knowledge integral to families' well‐being and collaborated with teachers to incorporate this knowledge into innovative teaching practices. Their research revealed that teachers who engaged with their students' family contexts gained a more holistic understanding of the child as an active participant across multiple knowledge domains and relational spheres. Early studies, as well as more recent ones (Moll & González, 1994; Da Silva Iddings, 2017), demonstrated that applying the Funds of Knowledge framework enables educators and students to challenge deficit‐based perspectives. This approach reshapes how literacy is used—as a tool for inquiry, critical thinking, and reflection on learning through new topics, activities, and questions.

Moll and González (1994) noted early on that linguistically diverse children strategically use language and literacy in their homes and communities to learn and communicate. They argued that “these children (and their communities) contain extensive resources, which they termed Funds of Knowledge, that can form the basis of an education that addresses broader social, academic, and intellectual issues than simply learning basic and rudimentary skills” (p. 441). Their studies revealed that linguistically diverse families employ print texts and oral and visual forms of communication—such as pictures, graphics, sign language, gestures, and drama—as part of their everyday activities. Children develop reading and writing skills through these multimodal engagements while accumulating essential knowledge, including numerical, digital, and multimodal literacy.

Over time, the Funds of Knowledge framework has illuminated the educational potential of minoritized populations, gaining traction globally as a transformative approach in educational research and practice. Teacher candidates in university‐based education programs often arrive with preconceived epistemologies and beliefs about language and diversity. A Funds of Knowledge perspective encourages these future educators to expand beyond their initial understandings, fostering growth through interactions with students, families, and communities. These experiences help prospective teachers reflect on how their practices influence culturally and linguistically diverse learners.

Teacher education programs that integrate university coursework with field experiences and foster connections between families, communities, and schools can offer richer learning opportunities. Such programs allow educators to better understand the lived experiences of their students across varied contexts. Immersion in these experiences not only prompts future teachers to question their existing beliefs but also supports the gradual transformation of their epistemologies regarding diverse learners. By prioritizing a Funds of Knowledge framework, teacher education programs can prepare educators to recognize, value, and draw upon the cultural wealth and knowledge of the communities they serve.

#### Bridging family‐school educational practices

In the U.S., numerous foundational studies have emphasized the importance of involving immigrant families in teacher education programs to counter widespread deficit‐based assumptions. In her influential book, Guadalupe Valdés (1996) explores parental involvement in U.S. schools and the efforts aimed at improving immigrant students' academic achievement through family‐oriented interventions. Valdés provides ethnographic insights into the daily lives of 10 immigrant families participating in a family literacy program, highlighting the need to bridge home and school experiences for immigrant students. She reveals that “parent education programs that are widely implemented in schools serve to foster a ‘why won't you be more like me’ mentality and thus maintain the status quo” (p. 113). While well‐intentioned, these programs often fail to view multiculturalism as an asset, misinterpreting immigrants' cultural characteristics as disinterest in their children's education. Valdés underscores that immigrant families, such as those of Mexican blue‐collar backgrounds, bring unique goals, experiences, and life plans that may not align with the expectations of school culture or definitions of success. She argues that the lack of student achievement gains observed in these families is not due to poor parenting but to programs that seek to change families without respecting or valuing their sociocultural frameworks.

Similarly, Concha Delgado‐Gaitán (2013) offers a theory of literacy as a social practice deeply tied to the family's organizational structure. Delgado‐Gaitán considers the intricate cultural fabric of immigrant families, including their socioeconomic status, political relationships, religious beliefs, and connections with institutions like schools and neighborhoods. Her work illustrates how family literacy programs can empower parents by granting them access to literate communities and enabling them to interpret texts through their lived experiences. Empowerment, as defined by Delgado‐Gaitán, involves “the ability to influence intended and unintended effects for oneself and others, and the increased capacity to create the desired change that the individual, parents, family, teacher, and school deem appropriate” (p. 146). She highlights that empowerment at the individual level fosters self‐esteem and confidence, laying the groundwork for systemic change.

The Funds of Knowledge and Identity approach has only recently been applied to curriculum and instruction in Chile. D'warte and Woodrow (2023) conducted research in 20 early childhood education centers in communities with high levels of poverty and limited family involvement in children's learning over a 6‐year period. Their findings revealed significant physical and institutional barriers that prevented families from engaging in the pedagogical process. These barriers, stemming from the vulnerabilities and insecurities experienced by these communities, resulted in a lack of meaningful curricular spaces for participation. However, by incorporating artistic strategies and literary cafés, families began sharing their knowledge of child‐rearing and survival, enriching the official curriculum. These changes followed a training phase for educators, which helped shift beliefs about the so‐called “deficit” cultures of families from marginalized communities.

Further research in Chile has demonstrated the transformative potential of the Funds of Knowledge approach. Luna et al. (2023) analyzed its application in culturally and ethnically diverse schools, allowing children to explore their own Family Funds of Knowledge. By applying the method's techniques in their homes, children and teachers reported a strengthened recognition and valuation of their cultural roots, improved familial ties, and enhanced peer relationships, fostering coexistence. Similarly, Muñoz et al. (2022) examined the pedagogical potential of Funds of Knowledge in Mapuche communities. Their findings highlighted core aspects of the Mapuche cultural matrix—such as family ancestry, territorial origins, language, and spirituality—that can contextualize education and challenge colonialist, Eurocentric school traditions. However, these opportunities are often constrained by the colonial foundations of the educational system, which resist such transformative practices.

### Research illustrations for the conceptual application of funds of knowledge in teacher education programs

To address our central research question, we offer illustrations from two larger research projects conducted in Chile and the U.S., each aimed at supporting pre‐and in‐service teachers in developing a deeper understanding of immigrant families. These understandings were informed by the families’ histories, experiences of displacement, relationships with their communities, and interactions with schools. By focusing on these dimensions, the projects challenged the persistent deficit narratives surrounding immigrant students and families in both countries. The concept of Funds of Knowledge provided the analytical framework for these studies, guiding innovative curricular and field experiences designed to prepare educators to work more effectively with immigrant students and their families.

### Methodological overview of projects

For both projects, the authors used a participatory ethnographic methodological approach aimed to access the real‐life contexts of families' educational practices respectfully and rigorously, offering a comprehensive understanding of their Funds of Knowledge (see McWayne, Melzi & Mistry, 2022). This approach combined verbal and visual techniques to provide diverse forms of expression for the participating families (Esteban‐Guitart et al., 2022). Three primary techniques included: In‐depth interviews conducted at the beginning and end of the projects, family genograms, which participants drew to map relationships and histories, and photographic analysis of educational routines, where families documented their daily activities through photographs and later discussed these with researchers during home visits.

In both contexts, preservice teachers engaged in reflective coursework before conducting periodic visits to immigrant families' homes. These visits, spanning several months, prepared teachers to adopt a curious and open‐minded stance, suspend preconceived notions, and act as learners of the families' social, cultural, and linguistic practices. During these visits, preservice teachers used the Funds of Knowledge protocol (González et al., 2005) alongside emergent interview questions to explore the households' cultural and educational practices. Post‐visit debriefings allowed for in‐depth discussions of their observations, understandings, and biases, fostering critical reflection.

### Illustrations of funds of knowledge applications in the U.S. and in Chile

Fund of Knowledge was applied in two unique contexts: The U.S.‐based project focused on redesigning an undergraduate early childhood teacher education program. Teacher candidates were required to interact directly with immigrant families—often in their homes—to learn about their language and literacy practices. These experiences allowed candidates to reflect on their findings and integrate the families' cultural and linguistic practices into their teaching, seeing immigrant households as rich sources of knowledge and using this understanding to inform curriculum development and teaching strategies (Da Silva Iddings, 2017). The Chile‐based project incorporated Funds of Knowledge as a foundational concept in undergraduate and graduate university courses to prepare educators to work with immigrant populations. Initial teacher interviews explored how educators currently include immigrant families' Funds of Knowledge in the curriculum to identify areas of continuity and discontinuity between family practices and school‐based education. This project demonstrated the transformative potential of integrating families' knowledge into the school curriculum.

### Application of funds of knowledge in teacher education in the U.S

This section describes the redesign and implementation of a university‐based early childhood teacher education program grounded in the Funds of Knowledge framework (Da Silva Iddings, 2017).

#### Design

The program's redesign aimed to amplify the voices of immigrant families, who served as partners in its conceptualization. A problem‐solving approach, focusing on addressing real‐world challenges through the application of local knowledge contextualized within community settings, characterized this program. Through collaborative interactions among families, teachers, university researchers, and preservice teachers, we developed a teacher preparation curriculum focusing on culturally responsive teaching methods. Key to the program activities was the establishment of designated spaces for these collaborations to flourish and take hold so that educational experiences that were designed were fundamentally guided by families' contributions. In addition, we understood that a strong commitment to long‐term and sustained engagement with families and communities was necessary to build trusting relationships. A sustainability plan was put into effect continuously in the design as we focused on long‐term implementation and scalability.

#### Implementation

Following the program's redesign, a research team composed of two university professors and two graduate students conducted a 2‐year study to evaluate its implementation. The study employed a multiple case study methodology, collecting and analyzing a diverse array of data, including field notes, video and audio recordings, and samples of children's emergent literacy documented during family home interactions. During these home interactions, prospective teachers observed and documented children's language development and family literacy practices. These observations provided critical insights into the cultural and educational practices of immigrant families.

Focusing on how interactions between prospective teachers and immigrant families influenced the teachers' understanding of Funds of Knowledge and cultural practices, key areas of analysis included: reflections by prospective teachers, examining how engagement with families shaped the teachers' attitudes and deepened their appreciation for immigrant households' cultural and linguistic richness and analysis of literacy interactions focusing on how activities with families facilitated an understanding of the diverse ways immigrant children develop language and literacy skills in their home environments.

Through this collaborative and immersive approach, the program enabled future educators to move beyond deficit‐oriented perspectives and embrace a more nuanced, respectful, and culturally informed approach to teaching. In the redesigned early childhood teacher education program, each candidate had to conduct at least 12 home interactions during their final 2 years. In collaboration with family members, candidates also co‐designed and co‐implemented social and literacy initiatives for school and community families in various formats. These activities aimed to recognize young children's cultural backgrounds, build on families' language and literacy histories, promote social interactions and community leadership among parents, and develop shared literacy practices for use in classrooms and at home.

These home‐based engagements provided crucial insights into how children navigate their multiple communities, adjust to school environments, and begin acquiring the formal language of education. By observing children and their families, teacher candidates gained a nuanced understanding of the creative strategies and skills families employ as they adapt to new linguistic and cultural contexts. This process enabled candidates to comprehend better how children develop multilingualism, multiple literacies, and cultural competencies rooted in their families' practices.

#### Evaluation

To evaluate the effectiveness of the redesigned program, in‐depth interviews were conducted with graduates from five cohorts of preservice teachers (approximately 150 participants). These interviews explored shifts in deficit‐oriented mindsets, particularly concerning the hegemonic English language ideologies and their impact on teaching practices, the perceived role of families in their children's education, and families' emphasis on literacy development, and their cultural and linguistic contributions. Graduates consistently reported that the home‐based engagements were transformative. They expressed that these experiences significantly enhanced their understanding of the families' roles in children's education and provided practical insights into language and literacy practices, as the following excerpt from one of our teacher candidates illustrates:

“I think just to have seen a family value literacy above many other things and consider it more important than other things in their life has been interesting. I have seen how much her desire to learn has changed, considering she is almost 4 years old…she just turned four…and she is already reading. That is a very important thing.” Amanda.

In addition, our teacher candidates came to understand home interactions as a space where they can learn from and with students and their families about their everyday language and literacy practices through conversations, joint projects, and family and community interactions (González et al., 2005). Then, based on this understanding, teacher trainees were able to critically reflect and integrate their new perspectives into their instructional planning of activities. To this end, we provide below an excerpt from an interview with Sarah:

“I think, in particular, knowing things about the kids in my class, their backgrounds and their families, like where their parents or their parents' parents are from and what they know about that culture and that part of the world. It helps you. It helps you. You can create a lot of learning opportunities and lessons in the classroom based on that idea or the things that they know.”

These curricular activities allowed teacher candidates to critically examine their assumptions about immigrants and the families they work with and engage with their knowledge base to successfully integrate them into the school curriculum. Overall, in this study, we found that the preservice teachers' extensive and consistent experiences interacting with families over two academic years allowed them to reflect on how their views of those interactions had evolved. In all cases, the teacher candidates we interviewed commented on how they appreciated interacting with children, their parents, and their family in a variety of nonschool contexts. For these teacher candidates, gradual, uneven, and continuous learning about the Funds of Knowledge challenged the ways and possibilities of adopting this perspective as part of their professional experience.

As teacher educators and researchers, using a Funds of Knowledge approach as a fundamental tenet of our early childhood teacher education program allowed us to counter the still prevalent ideology that culturally and linguistically diverse students and their families are somehow deficient in language and literacy or lack such resources (i.e., a deficit‐oriented perspective). Our redesigned program, and specifically the field experiences, relied on the ability of prospective teachers to identify and tap into those Funds of Knowledge that each of them, students, and teachers alike, bring to the classroom from their family and home experience.

### The application of funds of knowledge in teacher education in Chile

In the case of Chile, the objectives of the program were to learn about the relevant educational practices of migrant families in relation to their Funds of Knowledge, contrasting them with the school educational practices for the inclusion of foreign students of current teachers to identify points of convergence and elements of continuity between families and schools that would allow the initiation of design activities. At the same time, the aim was to identify issues that hindered teachers from incorporating culturally responsive teaching and/or working from an intercultural perspective.

#### Design

A team of psychologist and sociologist researchers visited the homes of the families that participated in the project, a total of 12 families of Venezuelan origin and one family of Colombian origin. The data collection method took elements of the ethnographic approach, whose main data collection technique is participant observation. Of particular interest was to gain access in a rigorous and respectful manner to household activities where relevant educational practices took place, and then to obtain a deep and as complete as possible understanding of these practices. For this reason, frequent visits were made to the homes of the migrant families for 2 months. Information production techniques with participatory and qualitative characteristics were used, combining verbal and visual techniques to offer different ways of expression to the participants.

Three techniques were chosen for the project: the in‐depth interview (at the beginning and end of the information‐gathering process), the drawing of the family genogram of at least three previous generations and its analysis together with the families, where topics related to jobs and trades of parents and grandparents, transgenerational importance attributed to schooling, relationship, and forms of communication with the school were investigated. We also used the analysis of educational routines through photographs taken by the families during the week. These photographs were then discussed with the researchers. Parents were asked to take photographs during the week of those daily situations in which they thought they were teaching their children something.

#### Implementation

At least five visits were made to the families' homes, each lasting approximately 2 h, using the techniques per session and a final session to validate the results with them. Observations were recorded through field notes. Content analysis was used for the treatment of the data obtained, through predefined categories related to the identification of the main Funds of Knowledge, the transgenerational relationship with the school, and expectations regarding the current migratory context. At the same time, interviews were conducted with the teachers who assisted these families and the children in their respective schools, to learn about the teachers' educational practices for the inclusion of immigrant students, to contrast elements of continuity and discontinuity between family and school knowledges. In addition, the academic expectations for the students in their new school were identified.

These findings were discussed with the teachers, and together with the researchers, they contemplated several scenarios of possibilities to create classroom activities that considered not only the families' knowledge and skills but also their high motivation to participate in the new school and to make known their own way of being and feeling. Institutional restrictions that constrained the design of the activities were also considered. The last phase of creating classroom curricular activities was the elaboration stage, which counted with the participation of undergraduate and graduate students in sociology and psychology.

#### Evaluation

Analyses of family practices revealed the development of a sophisticated and deeper knowledge of immigrant families' practices, as well as a clearer continuity between this knowledge, families' goals and practices, and the expectations, attitudes, and behaviors at the schools. Continuities between family, community, and school practices are possible to the extent that immigrant families have often internalized, transgenerationally, certain social conventions about how to function, relate, and behave within the school culture. To illustrate this, we offer an excerpt from one of the interviews conducted with a mother participating in the project:

“I tell her that she (the daughter) has to study; her duty is to study and listen to the teacher. I tell her, the mother takes care of you, she makes you food, she spoils you, and the only thing you have to do is do your homework and get good grades.”

These social conventions are often consistent with school practices and objectives. They are highly valued by teachers, contradicting culturally ingrained beliefs about the social and educational deficit of Latin American immigrant families in Chile. To illustrate this, we point to an excerpt from interviews conducted with a teacher in the urban area:

“Not to make comparisons because one should never make comparisons, but the children who come from Venezuela, for example, have a much higher level, at least in mathematics. They express themselves much better, they have a much wider vocabulary. And the rules of education, silence, respect, is something that comes from their families”.

In addition, these studies have found that these immigrant students progress much faster academically than Chilean students. This fact could be explained by the high expectations that parents have of their children in terms of both social and academic skills, since most parents coming from Latin America to Chile have completed their formal education and, in some cases, have a remarkably high level of education, some with postgraduate degrees. In addition, these studies found that immigrant children from Latin America tend to have a much richer vocabulary than Chilean students enrolled in schools classified as vulnerable. All this suggests incorporating the Funds of Knowledge perspective with Chilean students and their families (Lamas Aicón & Thibaut, 2021). Venezuelan students have enormously contributed to their host educational contexts in these and many other aspects. At least in the rural school, their arrival has meant for the other students an opportunity to learn from other cultures and latitudes, showing much interest in the newcomers, as illustrated in the following excerpt from an interview with a teacher at this school:

“I think they come in here almost like rockstars. I have not seen the Venezuelan kids, none of them being discriminated against… and it shows, because in fact the kids have a good relationship in general with their classmates. And they listen to them because (the Venezuelan boy), he is president of the class, but also of the general student center. A Venezuelan! And he went to talk even with President Boric, he took pictures, he even introduced him to his classmates, these students have a lot of personality, and the other kids admire them”.

However, these high capacities mentioned in the quotes above may be perceived and valued by schools from a utilitarian point of view, that is, in line with the characteristics of functional interculturality (Lamas‐Aicón et al., 2023) since they are families that effectively help schools to achieve the academic results that the system demands from an assimilationist point of view, and not from the examination of hegemonic, racialized practices and power relations within the school. In other words, the structures remain intact, even reinforced, and the transformation of the curriculum regresses. In this sense, the question arises as to how best to use the concept of Funds of Knowledge in university courses to prepare educators to understand the linguistic and cultural practices of immigrant families and investigate and problematize the systemic oppression.

### Cross‐cutting themes across contexts

The case studies presented here approach the concept of Funds of Knowledge as foundational in teacher education university programs, coursework, and field experiences. Through these illustrations, we argue the importance of engaging educators in thinking critically about systemic and structural issues that perpetuate oppression, illegitimacy, and exclusion of immigrant students and families. In addition, we highlight the need to provide future teachers with ample opportunities to learn from the lived experiences of immigrant families and communities and to understand and reflect on their own Funds of Knowledge vis‐à‐vis those of their students, and to examine how these interact with pedagogical knowledge.

Although the concept of Funds of Knowledge was first developed to address home–school discontinuities for immigrant families living in the borderlands between the U.S. and Mexico, its application has shown to be relevant in various parts of the U.S. and the world. However, we point out that this concept's application must be mindful of and adapted according to the geographical, social, cultural, and historical contexts of practice. In broad strokes, for the two national contexts, the U.S. and Chile, implicated in our respective projects presented here, some similarities/differences are noted below:

#### The history of colonization

Despite differences in the histories of colonization in Chile and the U.S., in both contexts, perhaps to a different degree, there is a colonial matrix of power inherited, recreated and imposed on society and its institutions that maintain asymmetrical relations and hierarchical and racialized structures of one group over others in an ideology of homogenization and whitening of national unity. The classic intersections between the categories of race, gender, and social class, remain unchanged to account for the structural and systemic inequalities that shape the presence of a disenfranchised migrant—an *other*—different, precarious, who must be made invisible, avoided, excluded. But this is not only exercised on the migrant but also on the indigenous, racialized, and/or impoverished national population (Lamas Aicón & Thibaut, 2021).

#### Eurocentrism

In Chile and the U.S., the experiences of discrimination, racism and xenophobia are more present in the Afro‐descendant and Indigenous populations. In Chile, to a larger extent than in the U.S., the phenomenon does not seem to be present in the same way for migrant groups coming from Europe that are recognized there as *foreigners* as opposed to migrants, who are associated with the Latin American population (Tijoux and Córdova, 2015). While the European population is implicitly recognized as having a sophisticated network of funds of knowledge, Latin American families must exhibit and demonstrate them.

#### Adult‐centeredness

Another relevant aspect that emerges from the studies in Chile, is those referring to the invisibilization of migrant childhoods from the adult‐centric view, generalized among agents working with children (Pavez‐Soto & Galaz, 2018). It is worrying to observe how the emotional and affective burden of childhoods is minimized, both in terms of the discrimination practices to which they are subjected from an early age, the scant attention paid to issues such as migratory mourning and the construction of identities in contexts of discrimination and racism, as well as the fact that experiences of school violence or bullying in schools are eclipsed. All this indicates the need to move towards identity funds as a counteractive approach.

#### Participatory approach

Another relevant aspect of differentiation between the original Funds of Knowledge research done in the borderlands in the U.S. and the studies presented here is methodological. While the original designs of Funds of Knowledge in the U.S. consider ethnographic work carried out by the teachers themselves who visit the homes of the families, in our respective designs, we have opted to generate alliances between academia and schools, being the researchers who carry out the ethnographic work or the teachers in university programs who, subsequently, report to the schools or the students who self‐report their Funds of Knowledge of their families (Da Silva Iddings, 2017; Lamas Aicón & Thibaut, 2021).

## DISCUSSION

A Funds of Knowledge approach to teacher education encourages dynamic and sensitive responses to ever‐changing educational contexts. This is particularly crucial as schools in the U.S., Chile, and globally, serve increasingly diverse populations with varied cultural and linguistic identities. To meet the challenge of providing inclusive and equitable education, we argue that antiracist and decolonial education, anchored in a Funds of Knowledge framework, should serve as a central value orientation in teacher preparation programs.

This orientation demands that schools go beyond effectiveness to become safe, healthy, and respectful environments for immigrant students and families. Teacher education programs must involve the entire educational community, recognizing families as foundational educators and key protagonists of plurilingualism and pluri‐culturalism. Such programs should aim to: establish collaborative partnerships among universities, families, communities, and schools; facilitate personal and collective reflection on socio‐political issues, such as immigration; challenge prejudices and stereotypes entrenched in discourse and culture; and promote access to information rooted in respect for human rights.

### Collaborative partnerships and cultural coherence

The establishment of these partnerships enables educators to consider the historical and cultural contexts that immigrant children bring to school, fostering a holistic understanding of family and community Funds of Knowledge. This coherence between educational practices and family/community contexts becomes particularly transformative in early childhood education. By creating spaces where children can express their cultural practices—whether current or ancestral—teachers can affirm students' identities and cultivate a sense of belonging within the school environment. When children see their cultures integrated into school practices and feel their traditions are valued, they are more likely to develop a strong connection to their educational environment. This recognition validates their identities while enriching the cultural fabric of the school, promoting mutual understanding among peers.

### Transforming hegemonic structures

A Funds of Knowledge perspective, which values local knowledge (ancestral, indigenous, and community), paired with a critical intercultural approach, can challenge hegemonic, hierarchical, and colonial structures in education (Melo, 2019; Pérez Ríos & Cárdenas Vera, 2020). Integrating school‐based (scientific) knowledge with traditional (local) knowledge opens spaces for joint reflection among teachers, families, and communities. These spaces provide opportunities to address critical issues such as racism and xenophobia, power dynamics within schools, and subordination of immigrant students and families Such reflective practices encourage the development of decolonial and counter‐hegemonic pedagogies that empower immigrant children and their families. These pedagogies affirm diverse identities and create a personalized, rather than standardized, approach to teaching and learning, and can provide avenues for the empowerment of migrant children and families worldwide. It can potentially dismantle deficit‐oriented views by legitimizing family and community knowledge as integral to formal education. By centering immigrant voices in curriculum design, pedagogy, and school decision‐making processes, this framework highlights the collective agency and wisdom of immigrant groups and communities. This perspective ensures that personal, family, and community identities are not only acknowledged but are also active contributors to educational processes. This inclusivity fosters a more just and equitable educational environment, where every participant—students, families, and educators—can feel valued and respected.

### Empowerment and collective agency

Collaborative partnerships formed through this framework, particularly those engaged in co‐designing curricula, can serve as catalysts for situated reflection on pressing issues such as stereotypes, prejudices, racism, and ethnocentrism. By thematizing the asymmetrical and racialized relations embedded within an inherited colonial matrix of power, these partnerships create space for participants to critically examine their positions within these structures and our roles in both constructing and deconstructing systemic inequities. Such reflections not only enhance awareness but also open possibilities for *joint action* to confront and dismantle these inequities collaboratively.

### Research limitations & future directions

Regarding the limitations of the studies, in the case of Chile, it is considered a limitation to focus only on the Venezuelan population, since Haitian, Colombian and Peruvian families are those reported in the literature as highly racialized. Similarly, relationships between race, language, and culture were not fully explored in the case of the U.S. study. Therefore, the need for continuing to explore these relationships in both Chile and the U.S. is evident. Also, the small size of the sample makes us take the findings with caution, even when we are dealing with multiple case designs of qualitative methodologies, whose interest is not generalization, but rather to delve deeper into the phenomenon.

Despite these limitations, through our comparative research on the application of Funds of Knowledge to prepare teachers to work with immigrant students and families in different national contexts, we can conclude that by aligning family practices with instructional goals, teachers were better able to foster a more inclusive and culturally responsive learning environment, bridging gaps between home and school. In both the U.S. and Chile, we noted that teacher preparation programs aiming to challenge deficit perspectives, encourage reflection, and promote the integration of immigrant families' cultural practices into curricula can pave the way for more equitable and effective educational practices for immigrant students.
